# Crystal structure of dimethyl 3,3′-[(3-nitro­phen­yl)methyl­ene]bis­(1*H*-indole-2-carboxyl­ate) ethanol monosolvate

**DOI:** 10.1107/S1600536814022296

**Published:** 2014-10-24

**Authors:** Hong-Shun Sun, Yu-Long Li, Hong Jiang, Ning Xu, Hong Xu

**Affiliations:** a Chemical Engineering Department, Nanjing College of Chemical Technology, Nanjing 210048, People’s Republic of China

**Keywords:** indole, crystal structure, MRI contrast agent

## Abstract

In the title compound, C_27_H_21_N_3_O_6_·C_2_H_5_OH, the indole ring systems are approximately perpendicular to each other, with a dihedral angle of 89.3 (5)°; the plane of the benzene ring is oriented with respect to the indole ring systems at 49.9 (5) and 73.4 (3)°. In the crystal, mol­ecules are linked by N—H⋯O and O—H⋯O hydrogen bonds and weak C—H⋯π inter­actions into a three-dimensional supra­molecular architecture. A void of 33.0 (7) Å^3^ is observed in the crystal structure. The solvent ethanol molecule acts as a donor, forming an O—H⋯O hydrogen bond, reinforcing the framework structure.

## Abstract 2

In the title compound, the planes of the two indole ring systems are approximately perpendicular to each other, with a dihedral angle of 89.3 (5)°.

## Chemical context   

Indole derivatives are found abundantly in a variety of natural plants and exhibit various physiological properties (Poter *et al.*, 1977 ▶; Sundberg, 1996 ▶). Among them, bis-indolymethane derivatives are found to be potentially bioactive compounds (Chang *et al.*, 1999 ▶; Ge *et al.*, 1999 ▶). In recent years, the synthesis and application of bis-indolymethane derivatives have been widely studied. The title compound is one of the bis-indolymethane derivatives as a precursor for MRI contrast agents (Ni, 2008 ▶). We report herein the synthesis and crystal structure of the title compound.
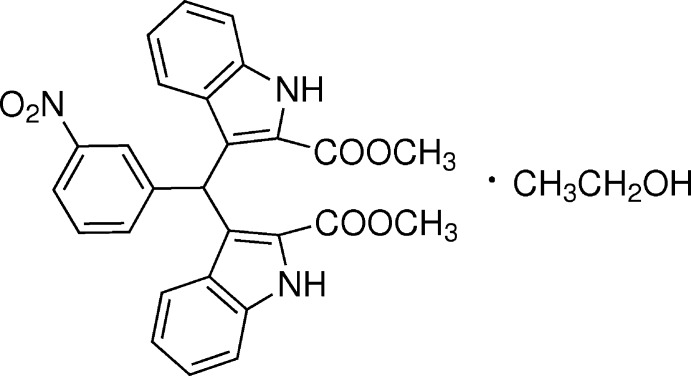


## Structural commentary   

**Figure 1:**
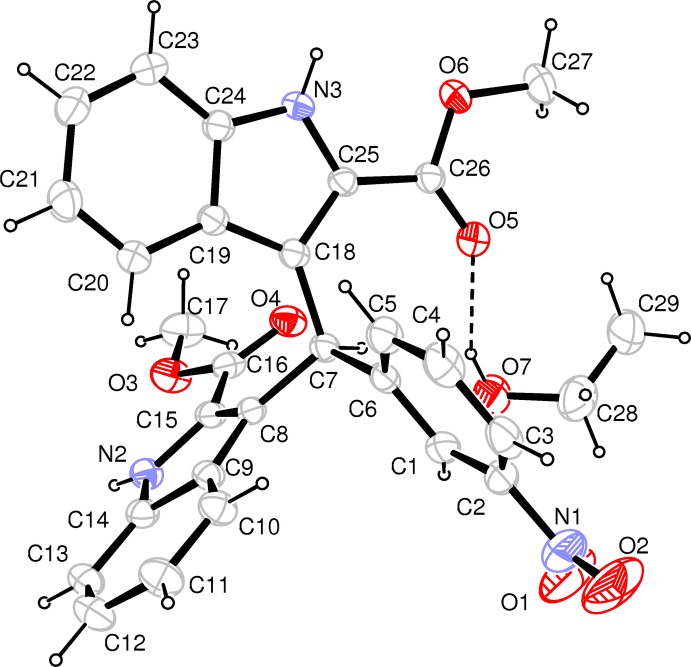
The mol­ecular structure of the title mol­ecule. showing the atom-labelling scheme. Displacement ellipsoids are drawn at the 30% probability level. The dashed line indicates the hydrogen bond between the main molecule and the ethanol solvent molecule.

The mol­ecular structure of the title compound is shown in Fig. 1 ▶. The two indole ring systems are nearly perpendicular to each other [dihedral angle = 89.3 (5)°] while the benzene ring (C1–C6) is twisted to the N1/C8–C15 and N2/C18–C25 indole ring systems with dihedral angles of 49.9 (5) and 73.4 (3)°, respectively. The carboxyl groups are approximately coplanar with the attached indole ring systems, the dihedral angles between the carboxyl groups and the mean plane of attached indole ring system are 10.0 (3) and 4.0 (4)°. The nitro group is also nearly coplanar with the attached benzene ring, the dihedral angle being 7.7 (7)°. A void of 33.0 (7) Å^3^ is observed in the crystal structure. The solvent ethanol molecule acts as a donor, forming an O—H⋯O hydrogen bond, reinforcing the framework structure.

## Supra­molecular features   

In the crystal, the organic mol­ecules and ethanol solvent mol­ecules are linked by classic N—H⋯O and O—H⋯O hydrogen bonds and weak C—H⋯π inter­actions involved the benzene rings, forming the three-dimensional supra­molecular architecture (Table 1 ▶).

## Database survey   

Several similar structures have been reported previously, *i.e.* diethyl 3,3′-(phenyl­methyl­ene)bis­(1*H*-indole-2-carboxyl­ate) (Sun *et al.*, 2012 ▶) and dimethyl 3,3′-(phenyl­methyl­ene)bis­(1*H*-indole-2-carboxyl­ate) (Sun *et al.*, 2013 ▶). In those structures, the two indole ring systems are also nearly perpendicular to each other, the dihedral angles are 82.0 (5) and 84.5 (5)°, respectively.

## Synthesis and crystallization   

Methyl indole-2-carboxyl­ate (17.5 g, 100 mmol) was dissolved in 200 ml methanol; commercially available 3-nitro­benzaldehyde (7.6 g, 50 mmol) was added and the mixture was heated to reflux temperature. Concentrated HCl (3.7 ml) was added and the reaction was left for 1 h. After cooling the white product was filtered off and washed thoroughly with methanol. The reaction can be followed by thin-layer chromatography (CHCl_3_–hexane = 1:1 *v*/*v*). The yield was 90%. Crystals of the title compound suitable for X-ray analysis were obtained by slow evaporation of an ethanol solution.

## Refinement   

H atoms were positioned geometrically, with N—H = 0.86Å and O—H = 0.82Å, and C—H = 0.93, 0.96, 0.97 or 0.98 Å for aromatic, methyl, methene and methine H atom, respectively, and constrained to ride on their parent atoms, with *U*_iso_(H) = *xU*_eq_(C,N,O), where *x* = 1.5 for methyl and hy­droxy, and *x* = 1.2 for all other H atoms.

## Supplementary Material

Crystal structure: contains datablock(s) I, global. DOI: 10.1107/S1600536814022296/xu5823sup1.cif

Structure factors: contains datablock(s) I. DOI: 10.1107/S1600536814022296/xu5823Isup2.hkl

Supporting information file. DOI: 10.1107/S1600536814022296/xu5823Isup3.cml

CCDC reference: 1028397

Additional supporting information:  crystallographic information; 3D view; checkCIF report

## Figures and Tables

**Table 1:** Hydrogen-bond geometry (, ) *Cg*3, *Cg*4 and *Cg*5 are the centroids of the C1-ring, C10-ring and C20-ring, respectively.

*D*H*A*	*D*H	H*A*	*D* *A*	*D*H*A*
N2H2*A*O7^i^	0.86	2.17	2.924(3)	146
N3H3*A*O4^ii^	0.86	2.02	2.861(4)	166
O7H7*B*O5	0.82	2.13	2.892(4)	154
C10H10*A* *Cg*3	0.93	2.87	3.633(4)	140
C11H11*A* *Cg*5^iii^	0.93	2.76	3.634(4)	156
C17H17*B* *Cg*4^i^	0.96	2.89	3.813(5)	163
C27H27*B* *Cg*5^ii^	0.96	2.75	3.496(4)	135

Symmetry codes: (i) 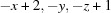; (ii) 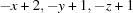; (iii) 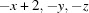.

**Table 2:** Experimental details

Crystal data	
Chemical formula	C_27_H_21_N_3_O_6_C_2_H_6_O
*M*_r_	529.54
Crystal system, space group	Triclinic, *P* 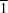
Temperature (K)	293
*a*, *b*, *c* ()	11.074(2), 11.585(2), 12.898(3)
, , ()	114.09(3), 106.68(3), 99.20(3)
*V* (^3^)	1372.5(5)
*Z*	2
Radiation type	Mo *K*
(mm^1^)	0.09
Crystal size (mm)	0.30 0.20 0.10

Data collection	
Diffractometer	EnrafNonius CAD-4
Absorption correction	scan (North *et al.*, 1968 ▶)
*T*_min_, *T*_max_	0.973, 0.991
No. of measured, independent and observed [*I* > 2(*I*)] reflections	5313, 5032, 3254
*R*_int_	0.029
(sin /)_max_ (^1^)	0.604

Refinement	
*R*[*F* ^2^ > 2(*F* ^2^)], *wR*(*F* ^2^), *S*	0.059, 0.166, 1.04
No. of reflections	5032
No. of parameters	352
H-atom treatment	H-atom parameters constrained
_max_, _min_ (e ^3^)	0.19, 0.26

Computer programs: *CAD-4 EXPRESS* (EnrafNonius, 1994 ▶), *XCAD4* (Harms Wocadlo, 1995 ▶) and *SHELXTL* (Sheldrick, 2008 ▶).

## supplementary crystallographic information

### Crystal data

**Table:**

C_27_H_21_N_3_O_6_·C_2_H_6_O	*Z* = 2
*M**_r_* = 529.54	*F*(000) = 556
Triclinic, *P*1	*D*_x_ = 1.281 Mg m^−^^3^
Hall symbol: -P 1	Mo *K*α radiation, λ = 0.71073 Å
*a* = 11.074 (2) Å	Cell parameters from 25 reflections
*b* = 11.585 (2) Å	θ = 9–13°
*c* = 12.898 (3) Å	µ = 0.09 mm^−^^1^
α = 114.09 (3)°	*T* = 293 K
β = 106.68 (3)°	Block, colorless
γ = 99.20 (3)°	0.30 × 0.20 × 0.10 mm
*V* = 1372.5 (5) Å^3^	

### Data collection

**Table:**

Enraf–Nonius CAD-4 diffractometer	3254 reflections with *I* > 2σ(*I*)
Radiation source: fine-focus sealed tube	*R*_int_ = 0.029
Graphite monochromator	θ_max_ = 25.4°, θ_min_ = 1.9°
ω/2θ scans	*h* = 0→13
Absorption correction: ψ scan (North *et al.*, 1968)	*k* = −13→13
*T*_min_ = 0.973, *T*_max_ = 0.991	*l* = −15→14
5313 measured reflections	3 standard reflections every 200 reflections
5032 independent reflections	intensity decay: 1%

### Refinement

**Table:**

Refinement on *F*^2^	Primary atom site location: structure-invariant direct methods
Least-squares matrix: full	Secondary atom site location: difference Fourier map
*R*[*F*^2^ > 2σ(*F*^2^)] = 0.059	Hydrogen site location: inferred from neighbouring sites
*wR*(*F*^2^) = 0.166	H-atom parameters constrained
*S* = 1.04	*w* = 1/[σ^2^(*F*_o_^2^) + (0.0769*P*)^2^ + 0.4188*P*] where *P* = (*F*_o_^2^ + 2*F*_c_^2^)/3
5032 reflections	(Δ/σ)_max_ = 0.001
352 parameters	Δρ_max_ = 0.19 e Å^−^^3^
0 restraints	Δρ_min_ = −0.26 e Å^−^^3^

### Special details

**Table:**

Geometry. All esds (except the esd in the dihedral angle between two l.s. planes) are estimated using the full covariance matrix. The cell esds are taken into account individually in the estimation of esds in distances, angles and torsion angles; correlations between esds in cell parameters are only used when they are defined by crystal symmetry. An approximate (isotropic) treatment of cell esds is used for estimating esds involving l.s. planes.
Refinement. Refinement of *F*^2^ against ALL reflections. The weighted *R*-factor *wR* and goodness of fit *S* are based on *F*^2^, conventional *R*-factors *R* are based on *F*, with *F* set to zero for negative *F*^2^. The threshold expression of *F*^2^ > σ(*F*^2^) is used only for calculating *R*-factors(gt) *etc*. and is not relevant to the choice of reflections for refinement. *R*-factors based on *F*^2^ are statistically about twice as large as those based on *F*, and *R*- factors based on ALL data will be even larger.

### Fractional atomic coordinates and isotropic or equivalent isotropic displacement parameters (Å^2^)

**Table:**

	*x*	*y*	*z*	*U*_iso_*/*U*_eq_	
N1	0.4712 (3)	−0.2518 (3)	0.0074 (4)	0.0901 (11)	
N2	1.1545 (2)	0.0075 (2)	0.4249 (2)	0.0438 (6)	
H2A	1.2151	0.0123	0.4870	0.053*	
N3	0.9859 (2)	0.4742 (2)	0.3448 (2)	0.0414 (5)	
H3A	0.9740	0.5507	0.3650	0.050*	
O1	0.4711 (3)	−0.2634 (3)	0.0977 (4)	0.1266 (13)	
O2	0.3872 (3)	−0.3273 (3)	−0.0959 (3)	0.1360 (14)	
O3	1.1911 (2)	0.21184 (19)	0.64357 (18)	0.0599 (6)	
O4	1.0053 (2)	0.25277 (18)	0.55999 (17)	0.0506 (5)	
O5	0.71167 (19)	0.2865 (2)	0.36509 (19)	0.0548 (5)	
O6	0.77081 (18)	0.49394 (18)	0.39092 (18)	0.0504 (5)	
O7	0.6624 (2)	0.0902 (2)	0.4450 (2)	0.0733 (7)	
H7B	0.7018	0.1489	0.4354	0.110*	
C1	0.6788 (3)	−0.0661 (3)	0.1405 (3)	0.0490 (7)	
H1A	0.6817	−0.0846	0.2047	0.059*	
C2	0.5754 (3)	−0.1429 (3)	0.0248 (3)	0.0565 (8)	
C3	0.5662 (3)	−0.1189 (3)	−0.0723 (3)	0.0616 (9)	
H3B	0.4956	−0.1715	−0.1484	0.074*	
C4	0.6637 (3)	−0.0153 (3)	−0.0543 (3)	0.0607 (9)	
H4A	0.6603	0.0025	−0.1190	0.073*	
C5	0.7674 (3)	0.0629 (3)	0.0598 (3)	0.0497 (7)	
H5A	0.8323	0.1337	0.0710	0.060*	
C6	0.7767 (3)	0.0382 (2)	0.1580 (2)	0.0395 (6)	
C7	0.8906 (2)	0.1240 (2)	0.2847 (2)	0.0361 (6)	
H7A	0.8495	0.1390	0.3451	0.043*	
C8	0.9896 (2)	0.0532 (2)	0.3134 (2)	0.0354 (6)	
C9	1.0194 (3)	−0.0546 (2)	0.2304 (2)	0.0389 (6)	
C10	0.9715 (3)	−0.1346 (3)	0.1015 (3)	0.0484 (7)	
H10A	0.9044	−0.1207	0.0497	0.058*	
C11	1.0243 (3)	−0.2330 (3)	0.0530 (3)	0.0598 (8)	
H11A	0.9910	−0.2870	−0.0321	0.072*	
C12	1.1276 (3)	−0.2544 (3)	0.1288 (3)	0.0609 (9)	
H12A	1.1624	−0.3211	0.0930	0.073*	
C13	1.1778 (3)	−0.1789 (3)	0.2542 (3)	0.0524 (8)	
H13A	1.2462	−0.1931	0.3044	0.063*	
C14	1.1231 (3)	−0.0798 (3)	0.3043 (2)	0.0410 (6)	
C15	1.0733 (3)	0.0872 (2)	0.4314 (2)	0.0390 (6)	
C16	1.0840 (3)	0.1913 (3)	0.5490 (2)	0.0425 (7)	
C17	1.2112 (4)	0.3165 (3)	0.7644 (3)	0.0695 (10)	
H17A	1.2900	0.3234	0.8255	0.104*	
H17B	1.1360	0.2952	0.7838	0.104*	
H17C	1.2208	0.3999	0.7635	0.104*	
C18	0.9553 (2)	0.2606 (2)	0.3046 (2)	0.0359 (6)	
C19	1.0709 (3)	0.3062 (2)	0.2854 (2)	0.0378 (6)	
C20	1.1640 (3)	0.2491 (3)	0.2473 (3)	0.0477 (7)	
H20A	1.1577	0.1615	0.2292	0.057*	
C21	1.2643 (3)	0.3253 (3)	0.2377 (3)	0.0624 (9)	
H21A	1.3258	0.2880	0.2123	0.075*	
C22	1.2763 (3)	0.4570 (3)	0.2648 (3)	0.0631 (9)	
H22A	1.3456	0.5054	0.2570	0.076*	
C23	1.1893 (3)	0.5166 (3)	0.3023 (3)	0.0529 (8)	
H23A	1.1980	0.6047	0.3208	0.064*	
C24	1.0863 (3)	0.4401 (3)	0.3120 (2)	0.0399 (6)	
C25	0.9066 (2)	0.3671 (2)	0.3405 (2)	0.0366 (6)	
C26	0.7876 (3)	0.3752 (3)	0.3664 (2)	0.0394 (6)	
C27	0.6529 (3)	0.5128 (3)	0.4122 (3)	0.0663 (9)	
H27A	0.6516	0.6011	0.4285	0.099*	
H27B	0.6530	0.5013	0.4819	0.099*	
H27C	0.5757	0.4485	0.3405	0.099*	
C28	0.5250 (4)	0.0591 (4)	0.3849 (4)	0.0870 (12)	
H28A	0.4800	−0.0202	0.3845	0.104*	
H28B	0.5049	0.0384	0.2998	0.104*	
C29	0.4715 (4)	0.1671 (5)	0.4421 (5)	0.1096 (17)	
H29A	0.3772	0.1388	0.3969	0.164*	
H29B	0.5131	0.2452	0.4404	0.164*	
H29C	0.4894	0.1872	0.5260	0.164*	

### Atomic displacement parameters (Å^2^)

**Table:**

	*U*^11^	*U*^22^	*U*^33^	*U*^12^	*U*^13^	*U*^23^
N1	0.062 (2)	0.059 (2)	0.101 (3)	−0.0028 (16)	0.000 (2)	0.025 (2)
N2	0.0404 (13)	0.0425 (13)	0.0432 (14)	0.0130 (11)	0.0046 (10)	0.0235 (11)
N3	0.0425 (13)	0.0313 (11)	0.0503 (14)	0.0153 (10)	0.0173 (11)	0.0187 (10)
O1	0.096 (2)	0.107 (3)	0.129 (3)	−0.0288 (19)	0.014 (2)	0.057 (2)
O2	0.102 (2)	0.087 (2)	0.109 (3)	−0.0398 (19)	−0.024 (2)	0.0179 (19)
O3	0.0643 (14)	0.0489 (12)	0.0396 (12)	0.0150 (10)	−0.0031 (10)	0.0140 (10)
O4	0.0582 (13)	0.0395 (11)	0.0457 (12)	0.0168 (10)	0.0147 (10)	0.0163 (9)
O5	0.0454 (12)	0.0523 (12)	0.0729 (15)	0.0170 (10)	0.0255 (11)	0.0333 (11)
O6	0.0448 (11)	0.0443 (11)	0.0645 (13)	0.0227 (9)	0.0227 (10)	0.0243 (10)
O7	0.0597 (14)	0.0859 (17)	0.0859 (17)	0.0228 (13)	0.0164 (13)	0.0598 (15)
C1	0.0440 (16)	0.0369 (15)	0.0531 (18)	0.0118 (13)	0.0097 (14)	0.0166 (13)
C2	0.0388 (16)	0.0382 (16)	0.064 (2)	0.0084 (13)	0.0055 (15)	0.0110 (15)
C3	0.0479 (19)	0.054 (2)	0.0476 (19)	0.0224 (16)	−0.0033 (15)	0.0054 (15)
C4	0.060 (2)	0.065 (2)	0.0404 (17)	0.0271 (18)	0.0054 (15)	0.0173 (15)
C5	0.0463 (17)	0.0507 (17)	0.0430 (17)	0.0149 (14)	0.0090 (14)	0.0203 (14)
C6	0.0372 (14)	0.0332 (14)	0.0385 (15)	0.0163 (12)	0.0081 (12)	0.0114 (12)
C7	0.0359 (14)	0.0326 (13)	0.0357 (14)	0.0115 (11)	0.0112 (11)	0.0142 (11)
C8	0.0331 (14)	0.0305 (13)	0.0385 (15)	0.0076 (11)	0.0090 (11)	0.0171 (11)
C9	0.0396 (15)	0.0355 (14)	0.0413 (15)	0.0124 (12)	0.0117 (12)	0.0207 (12)
C10	0.0544 (18)	0.0459 (16)	0.0425 (17)	0.0196 (14)	0.0143 (14)	0.0208 (14)
C11	0.077 (2)	0.0561 (19)	0.0492 (18)	0.0301 (18)	0.0282 (17)	0.0225 (15)
C12	0.073 (2)	0.0516 (18)	0.071 (2)	0.0364 (17)	0.0347 (19)	0.0298 (17)
C13	0.0521 (18)	0.0464 (17)	0.063 (2)	0.0234 (14)	0.0187 (16)	0.0296 (16)
C14	0.0419 (15)	0.0367 (14)	0.0438 (16)	0.0112 (12)	0.0134 (13)	0.0215 (13)
C15	0.0394 (15)	0.0315 (13)	0.0419 (15)	0.0086 (12)	0.0096 (12)	0.0189 (12)
C16	0.0462 (16)	0.0325 (14)	0.0395 (16)	0.0052 (13)	0.0062 (13)	0.0185 (12)
C17	0.086 (3)	0.0516 (19)	0.0377 (18)	0.0107 (18)	−0.0004 (17)	0.0122 (15)
C18	0.0348 (14)	0.0337 (13)	0.0319 (14)	0.0104 (11)	0.0062 (11)	0.0141 (11)
C19	0.0365 (14)	0.0350 (14)	0.0348 (14)	0.0113 (12)	0.0090 (12)	0.0139 (12)
C20	0.0458 (17)	0.0403 (15)	0.0557 (18)	0.0156 (13)	0.0210 (14)	0.0205 (14)
C21	0.053 (2)	0.063 (2)	0.074 (2)	0.0243 (17)	0.0356 (18)	0.0269 (18)
C22	0.0520 (19)	0.056 (2)	0.083 (2)	0.0128 (16)	0.0345 (18)	0.0313 (18)
C23	0.0534 (18)	0.0423 (16)	0.065 (2)	0.0129 (15)	0.0245 (16)	0.0278 (15)
C24	0.0390 (15)	0.0374 (14)	0.0397 (15)	0.0115 (12)	0.0118 (12)	0.0179 (12)
C25	0.0358 (14)	0.0320 (13)	0.0357 (14)	0.0108 (11)	0.0088 (11)	0.0141 (11)
C26	0.0351 (14)	0.0378 (15)	0.0394 (15)	0.0115 (12)	0.0085 (12)	0.0174 (12)
C27	0.0509 (19)	0.071 (2)	0.085 (3)	0.0347 (17)	0.0318 (18)	0.035 (2)
C28	0.062 (2)	0.098 (3)	0.091 (3)	0.010 (2)	0.005 (2)	0.059 (3)
C29	0.070 (3)	0.117 (4)	0.187 (5)	0.039 (3)	0.061 (3)	0.103 (4)

### Geometric parameters (Å, º)

**Table:**

N1—O2	1.218 (4)	C10—C11	1.369 (4)
N1—O1	1.226 (5)	C10—H10A	0.9300
N1—C2	1.459 (5)	C11—C12	1.404 (4)
N2—C14	1.362 (3)	C11—H11A	0.9300
N2—C15	1.383 (3)	C12—C13	1.364 (4)
N2—H2A	0.8600	C12—H12A	0.9300
N3—C24	1.358 (3)	C13—C14	1.395 (4)
N3—C25	1.372 (3)	C13—H13A	0.9300
N3—H3A	0.8600	C15—C16	1.458 (4)
O3—C16	1.339 (3)	C17—H17A	0.9600
O3—C17	1.456 (4)	C17—H17B	0.9600
O4—C16	1.214 (3)	C17—H17C	0.9600
O5—C26	1.211 (3)	C18—C25	1.385 (3)
O6—C26	1.337 (3)	C18—C19	1.433 (4)
O6—C27	1.441 (3)	C19—C20	1.409 (4)
O7—C28	1.402 (4)	C19—C24	1.414 (4)
O7—H7B	0.8200	C20—C21	1.372 (4)
C1—C6	1.380 (4)	C20—H20A	0.9300
C1—C2	1.393 (4)	C21—C22	1.393 (4)
C1—H1A	0.9300	C21—H21A	0.9300
C2—C3	1.370 (5)	C22—C23	1.359 (4)
C3—C4	1.369 (5)	C22—H22A	0.9300
C3—H3B	0.9300	C23—C24	1.397 (4)
C4—C5	1.384 (4)	C23—H23A	0.9300
C4—H4A	0.9300	C25—C26	1.457 (4)
C5—C6	1.388 (4)	C27—H27A	0.9600
C5—H5A	0.9300	C27—H27B	0.9600
C6—C7	1.529 (4)	C27—H27C	0.9600
C7—C18	1.511 (3)	C28—C29	1.482 (6)
C7—C8	1.521 (3)	C28—H28A	0.9700
C7—H7A	0.9800	C28—H28B	0.9700
C8—C15	1.384 (3)	C29—H29A	0.9600
C8—C9	1.435 (4)	C29—H29B	0.9600
C9—C10	1.408 (4)	C29—H29C	0.9600
C9—C14	1.420 (4)		
			
O2—N1—O1	122.4 (4)	N2—C15—C8	109.5 (2)
O2—N1—C2	118.9 (4)	N2—C15—C16	121.9 (2)
O1—N1—C2	118.7 (3)	C8—C15—C16	128.6 (2)
C14—N2—C15	109.2 (2)	O4—C16—O3	123.8 (3)
C14—N2—H2A	125.4	O4—C16—C15	123.9 (2)
C15—N2—H2A	125.4	O3—C16—C15	112.2 (2)
C24—N3—C25	109.1 (2)	O3—C17—H17A	109.5
C24—N3—H3A	125.4	O3—C17—H17B	109.5
C25—N3—H3A	125.4	H17A—C17—H17B	109.5
C16—O3—C17	115.9 (2)	O3—C17—H17C	109.5
C26—O6—C27	117.0 (2)	H17A—C17—H17C	109.5
C28—O7—H7B	109.5	H17B—C17—H17C	109.5
C6—C1—C2	118.6 (3)	C25—C18—C19	105.9 (2)
C6—C1—H1A	120.7	C25—C18—C7	124.8 (2)
C2—C1—H1A	120.7	C19—C18—C7	129.2 (2)
C3—C2—C1	122.8 (3)	C20—C19—C24	117.8 (2)
C3—C2—N1	118.8 (3)	C20—C19—C18	135.2 (2)
C1—C2—N1	118.3 (3)	C24—C19—C18	107.0 (2)
C4—C3—C2	118.2 (3)	C21—C20—C19	118.7 (3)
C4—C3—H3B	120.9	C21—C20—H20A	120.6
C2—C3—H3B	120.9	C19—C20—H20A	120.6
C3—C4—C5	120.2 (3)	C20—C21—C22	121.8 (3)
C3—C4—H4A	119.9	C20—C21—H21A	119.1
C5—C4—H4A	119.9	C22—C21—H21A	119.1
C4—C5—C6	121.5 (3)	C23—C22—C21	121.6 (3)
C4—C5—H5A	119.2	C23—C22—H22A	119.2
C6—C5—H5A	119.2	C21—C22—H22A	119.2
C1—C6—C5	118.6 (3)	C22—C23—C24	117.3 (3)
C1—C6—C7	119.3 (2)	C22—C23—H23A	121.4
C5—C6—C7	122.1 (2)	C24—C23—H23A	121.4
C18—C7—C8	113.3 (2)	N3—C24—C23	129.1 (2)
C18—C7—C6	112.2 (2)	N3—C24—C19	108.2 (2)
C8—C7—C6	113.1 (2)	C23—C24—C19	122.8 (3)
C18—C7—H7A	105.8	N3—C25—C18	109.9 (2)
C8—C7—H7A	105.8	N3—C25—C26	120.3 (2)
C6—C7—H7A	105.8	C18—C25—C26	129.7 (2)
C15—C8—C9	106.5 (2)	O5—C26—O6	123.4 (2)
C15—C8—C7	124.1 (2)	O5—C26—C25	125.2 (2)
C9—C8—C7	129.4 (2)	O6—C26—C25	111.4 (2)
C10—C9—C14	117.5 (2)	O6—C27—H27A	109.5
C10—C9—C8	135.7 (2)	O6—C27—H27B	109.5
C14—C9—C8	106.8 (2)	H27A—C27—H27B	109.5
C11—C10—C9	119.5 (3)	O6—C27—H27C	109.5
C11—C10—H10A	120.3	H27A—C27—H27C	109.5
C9—C10—H10A	120.3	H27B—C27—H27C	109.5
C10—C11—C12	121.5 (3)	O7—C28—C29	114.0 (4)
C10—C11—H11A	119.2	O7—C28—H28A	108.8
C12—C11—H11A	119.2	C29—C28—H28A	108.8
C13—C12—C11	121.1 (3)	O7—C28—H28B	108.8
C13—C12—H12A	119.4	C29—C28—H28B	108.8
C11—C12—H12A	119.4	H28A—C28—H28B	107.7
C12—C13—C14	117.7 (3)	C28—C29—H29A	109.5
C12—C13—H13A	121.2	C28—C29—H29B	109.5
C14—C13—H13A	121.2	H29A—C29—H29B	109.5
N2—C14—C13	129.2 (3)	C28—C29—H29C	109.5
N2—C14—C9	108.1 (2)	H29A—C29—H29C	109.5
C13—C14—C9	122.7 (3)	H29B—C29—H29C	109.5
			
C6—C1—C2—C3	−0.5 (4)	C7—C8—C15—N2	−177.3 (2)
C6—C1—C2—N1	−179.1 (3)	C9—C8—C15—C16	179.5 (3)
O2—N1—C2—C3	8.1 (5)	C7—C8—C15—C16	1.0 (4)
O1—N1—C2—C3	−171.5 (4)	C17—O3—C16—O4	−1.0 (4)
O2—N1—C2—C1	−173.3 (3)	C17—O3—C16—C15	178.6 (2)
O1—N1—C2—C1	7.1 (5)	N2—C15—C16—O4	−171.1 (2)
C1—C2—C3—C4	0.6 (5)	C8—C15—C16—O4	10.7 (4)
N1—C2—C3—C4	179.1 (3)	N2—C15—C16—O3	9.3 (4)
C2—C3—C4—C5	−0.7 (5)	C8—C15—C16—O3	−168.9 (3)
C3—C4—C5—C6	0.9 (5)	C8—C7—C18—C25	−149.2 (2)
C2—C1—C6—C5	0.6 (4)	C6—C7—C18—C25	81.3 (3)
C2—C1—C6—C7	179.7 (2)	C8—C7—C18—C19	34.6 (4)
C4—C5—C6—C1	−0.8 (4)	C6—C7—C18—C19	−95.0 (3)
C4—C5—C6—C7	−179.9 (2)	C25—C18—C19—C20	−178.9 (3)
C1—C6—C7—C18	−157.0 (2)	C7—C18—C19—C20	−2.1 (5)
C5—C6—C7—C18	22.1 (3)	C25—C18—C19—C24	0.5 (3)
C1—C6—C7—C8	73.4 (3)	C7—C18—C19—C24	177.3 (2)
C5—C6—C7—C8	−107.6 (3)	C24—C19—C20—C21	−0.2 (4)
C18—C7—C8—C15	73.3 (3)	C18—C19—C20—C21	179.1 (3)
C6—C7—C8—C15	−157.5 (2)	C19—C20—C21—C22	0.2 (5)
C18—C7—C8—C9	−104.8 (3)	C20—C21—C22—C23	0.0 (5)
C6—C7—C8—C9	24.3 (4)	C21—C22—C23—C24	−0.4 (5)
C15—C8—C9—C10	179.2 (3)	C25—N3—C24—C23	179.3 (3)
C7—C8—C9—C10	−2.4 (5)	C25—N3—C24—C19	0.2 (3)
C15—C8—C9—C14	−0.7 (3)	C22—C23—C24—N3	−178.7 (3)
C7—C8—C9—C14	177.7 (2)	C22—C23—C24—C19	0.4 (4)
C14—C9—C10—C11	0.8 (4)	C20—C19—C24—N3	179.1 (2)
C8—C9—C10—C11	−179.1 (3)	C18—C19—C24—N3	−0.4 (3)
C9—C10—C11—C12	−1.4 (5)	C20—C19—C24—C23	−0.1 (4)
C10—C11—C12—C13	1.1 (5)	C18—C19—C24—C23	−179.6 (3)
C11—C12—C13—C14	−0.1 (5)	C24—N3—C25—C18	0.1 (3)
C15—N2—C14—C13	−179.4 (3)	C24—N3—C25—C26	−177.3 (2)
C15—N2—C14—C9	0.8 (3)	C19—C18—C25—N3	−0.4 (3)
C12—C13—C14—N2	179.7 (3)	C7—C18—C25—N3	−177.4 (2)
C12—C13—C14—C9	−0.5 (4)	C19—C18—C25—C26	176.8 (2)
C10—C9—C14—N2	−180.0 (2)	C7—C18—C25—C26	−0.2 (4)
C8—C9—C14—N2	−0.1 (3)	C27—O6—C26—O5	−2.3 (4)
C10—C9—C14—C13	0.2 (4)	C27—O6—C26—C25	177.0 (2)
C8—C9—C14—C13	−179.9 (3)	N3—C25—C26—O5	−179.9 (2)
C14—N2—C15—C8	−1.2 (3)	C18—C25—C26—O5	3.2 (4)
C14—N2—C15—C16	−179.7 (2)	N3—C25—C26—O6	0.8 (3)
C9—C8—C15—N2	1.2 (3)	C18—C25—C26—O6	−176.1 (2)

### Hydrogen-bond geometry (Å, º)

Cg3, Cg4 and Cg5 are the centroids of the C1-ring, C10-ring and C20-ring, respectively.

**Table:**

*D*—H···*A*	*D*—H	H···*A*	*D*···*A*	*D*—H···*A*
N2—H2*A*···O7^i^	0.86	2.17	2.924 (3)	146
N3—H3*A*···O4^ii^	0.86	2.02	2.861 (4)	166
O7—H7*B*···O5	0.82	2.13	2.892 (4)	154
C10—H10*A*···*Cg*3	0.93	2.87	3.633 (4)	140
C11—H11*A*···*Cg*5^iii^	0.93	2.76	3.634 (4)	156
C17—H17*B*···*Cg*4^i^	0.96	2.89	3.813 (5)	163
C27—H27*B*···*Cg*5^ii^	0.96	2.75	3.496 (4)	135

Symmetry codes: (i) −*x*+2, −*y*, −*z*+1; (ii) −*x*+2, −*y*+1, −*z*+1; (iii) −*x*+2, −*y*, −*z*.
